# Scientists’ personality, values, and well-being

**DOI:** 10.1186/s40064-016-2225-2

**Published:** 2016-05-12

**Authors:** Wataru Sato

**Affiliations:** Department of Neurodevelopmental Psychiatry, Habilitation and Rehabilitation, Graduate School of Medicine, Kyoto University, 54 Shogoin-Kawaharacho, Sakyo, Kyoto, 606-8507 Japan

**Keywords:** Personality, Purpose in life, Scientist, Subjective happiness, Subjective well-being, Values

## Abstract

**Background:**

Scientists play an important role in modern society. However, only a small number of their psychological characteristics, such as personality traits, have been investigated; hence, further investigation is required.

**Results:**

In this study, scientists (*n* = 24) and non-scientist controls (*n* = 26) were assessed with respect to their five-factor personality traits, 10 basic values, and subjective well-being (subjective happiness and sense of purpose in life). Compared with the non-scientist control group and with normative data of laypeople, the scientists consistently exhibited greater openness (i.e., traits related to curiosity and intelligence), self-direction (i.e., values related to the pursuit of curiosity, creativity, and autonomous action), happiness, and sense of purpose in life.

**Conclusions:**

These data indicate that scientists possess personality traits and values suitable for a career in science, from which they also derive subjective well-being.

**Electronic supplementary material:**

The online version of this article (doi:10.1186/s40064-016-2225-2) contains supplementary material, which is available to authorized users.

## Background

Scientists play an important role in modern society. Based on scientific knowledge, technology has developed sufficiently such that present-day laypeople can lead convenient and prosperous lives. Simultaneously, scientific activities have occasionally produced dangerous scenarios, such as the creation of nuclear bombs that hold the potential to destroy the entire world. Therefore, society should maintain an appropriate level of understanding and control in relation to the activities of scientists.

However, the scientists’ psychological characteristics remain poorly understood compared with other aspects of information about them, such as historical accounts and philosophical foundations (Feist 2006b; Feist and Gorman 1998). Psychological research on scientists is important, as it has the potential to unveil their actual characteristics and may have implications for other literatures focused on the study of scientific disciplines (Shadish et al. 1989). Understanding the psychological characteristics of scientists may also prove useful in improving education and recruitment programs (Feist and Gorman 1998; Holland 1985; Houts 1989).

Among psychological characteristics, several studies have investigated the personality of scientists. Personality refers to stylistic and habitual patterns of affect, behavior, and cognition that can be measured reliably and validly using the five factors model (Pytlik et al. 2002). Previous studies have reported that scientists, specifically those involved in creative endeavors, exhibit high scores on the personality trait of openness (e.g., Lounsbury et al. 2012; Grosul and Feist 2014; for a review, see Feist 2006c). Based on the definition of openness in the five factors model (Barrick and Mount 1991), the data suggest that scientists possess imaginative, cultured, curious, original, broad-minded, intelligent, and artistically sensitive traits. Such information would be useful in improving the understanding of scientists’ psychology. It has also been pointed out that further research is necessary pertaining to other psychological aspects of scientists, such as their motivations (Feist 1998) and mental health (Feist 2006a).

The aim of the present study was to extend the understanding of the psychology of scientists. Data from a sample of scientists were collected using questionnaires. These data were then compared with those from the non-scientist control group and from normative data of laypeople. The personality traits of scientists were analyzed to confirm the previous findings. I predicted that scientists would exhibit greater openness compared with non-scientists.

In addition, the scientists’ values were investigated to determine their motivations. Previous studies have reported strong work motivation in scientists (e.g., McClelland 1962; for a review, see Feist 1998). However, where such motivations are directed remains unclear. This question was addressed in this study by assessing scientists’ values, that is, the basic motivations that guide their affect, behavior, and cognition (Schwartz 1992). I used Schwartz’s 10 basic values model (Schwartz 1992), which has been shown to be reliable, valid, and universally applicable (Schwartz 2012). Scholars have traditionally proposed that curiosity and intellectual exploration (Weber 1922) and autonomy/freedom (Polanyi 1958) are indispensable elements of scientists’ activities, although these claims are subject to debate (Broad and Wade 1982). Consistent with the traditional view, a qualitative interview study reported that scientists frequently referred to curiosity, the desire to conduct good science, and striving for self-fulfillment as their primary motivations (Jindal-Snape and Snape 2006). According to Schwartz’s 10 basic values model, self-direction, that is, the value placing importance on the pursuit of curiosity, creativity, and autonomous action (Schwartz 2012), is the value most closely connected to these self-reports. Based on these data, I predicted that scientists would exhibit higher levels of self-direction values than non-scientists.

Furthermore, the subjective well-being of scientists was investigated. Subjective well-being represents a positive aspect of mental health and has been proposed as being the ultimate goal of humans by several scholars, including Aristotle. A philosopher (Russell 1930) has proposed that scientific occupation is one of the most effective in eliciting feelings of happiness. Consistent with this idea, numerous anecdotal reports indicated that scientists acquire great happiness and satisfaction from their work (e.g., Wolpert and Richards 1997). Empirical studies have also shown that scientists are less likely to suffer from mental illness compared with non-scientists (Ludwig 1995; Rawlings and Locarnini 2008). However, no study to date has measured scientists’ subjective well-being. Based on the dominant theory that subjective well-being is multi-faceted, including both affective and eudaimonic components (Baumeister et al. 2013; Kauppinen 2013; Kringelbach and Berridge 2010), measures of subjective happiness (Lyubomirsky and Lepper 1999) and measures of sense of purpose in life (Crumbaugh and Maholick 1964) were implemented in this study. Based on the aforementioned literature, I predicted that scientists, as compared with non-scientists, would exhibit higher scores on these subjective well-being measures.

## Methods

### Participants

The scientist sample group consisted of 24 scientists (5 females, 19 males; mean ± SD age = 34.2 ± 4.2 years). All scientists were non-tenured associate or assistant professors at the Hakubi Center for Advanced Research, Kyoto University, which provides accommodation and support for multi-disciplinary researchers. Their fields of research were natural sciences (*n* = 21) and the humanities/social sciences (*n* = 3), according to their own categorizations chosen from among the options of natural science, humanities/social sciences, and art. All scientists held doctoral degrees.

For purposes of comparison with the scientists’ data, two data sets were prepared. First, data from a control group of 26 non-scientists (6 female, 20 males; mean ± SD age = 34.7 ± 4.5 years) were collected using the same questionnaires used for the scientists. All participants in the non-scientist control group were matched with the scientist group for age (independent *t* test, *p* < 0.01) and sex (χ^2^ test, *p* < 0.01). Their occupations, based on their own categorizations, included: office worker (*n* = 9), public service worker (*n* = 3), manufacturing worker (*n* = 3), cosmetic service provider (*n* = 2), domestic worker (*n* = 2), military force recruit (*n* = 2), builder (*n* = 1), mechanic (*n* = 1), medical service provider (*n* = 1), driver (*n* = 1), and part-time laborer (*n* = 1). Second, a large data set assembled from previous studies of Japanese adult laypeople was analyzed as normative data. Specifically, the data on personality, values, subjective happiness, and purpose in life were derived from the NEO Five Factor Inventory (NEO-FFI; Success Bell, Tokyo, Japan) (*n* = 328–334), Soldner (2013) (*n* = 164), Shimai et al. (2004) (*n* = 302), and Sato (1986) (*n* = 163).

The first language of all participants in both the scientist and non-scientist groups was Japanese. All participants provided informed consent after the experimental procedures were explained in full. This study was approved by the Ethics Committee of the Primate Research Institute, Kyoto University, and was conducted in accordance with the Declaration of Helsinki.

### Psychological questionnaires

Participants’ personality traits were assessed using the Japanese version of the NEO-FFI (Success Bell, Tokyo, Japan), a 60-item questionnaire that measures the five basic personality traits (12 items for each dimension) of neuroticism, extraversion, openness, agreeableness, and conscientiousness (e.g., for openness: “I have a lot of intellectual curiosity”). Each factor is described in Additional file 1: Table S1 according to Barrick and Mount (1991). The reliability and validity of the NEO-FFI has been verified previously in Japanese participants, according to the manual.

Participants’ values were assessed using the Japanese version of the portrait values questionnaire (PVQ) (Schwartz et al. 2001), a 40-item instrument assessing goals, aspirations, and desires that points implicitly toward the importance of values (e.g., for the self-direction value: “Thinking up new ideas and being creative is important to him. He likes to do things in his own original way”). The values are described in full in Additional file 1: Table S1 according to Schwartz (2012). The reliability and validity of the PVQ has been verified previously in Japanese participants (Soldner 2013).

To measure subjective happiness, the Japanese version of the subjective happiness scale (SHS) (Lyubomirsky and Lepper 1999; Shimai et al. 2004), a four-item questionnaire assessing global subjective happiness (e.g., “In general, I consider myself: not a very happy person/a very happy person”), was used. The reliability and validity of the SHS has also been verified in Japanese participants (Shimai et al. 2004).

Sense of purpose in life was assessed using the Japanese version of the purpose in life (PIL) test (Success Bell, Tokyo, Japan) (Crumbaugh and Maholick 1964), a 20-item questionnaire measuring the degree of existential meaning (e.g., “I am usually: completely bored/exuberant, enthusiastic”). As in the case of the other instruments, the reliability and validity of the PIL has been verified previously in Japanese participants, according to the manual.

### Data analysis

Scores on the NEO-FFI, PVQ, SHS, and PIL were calculated according to the instructions in their manuals or the methods employed in previous studies. Independent *t* tests were conducted to compare the scientist and non-scientist groups, and to compare the scientist group against the normative data. For the measures of interest described in the “Background” section, one-tailed tests were conducted. For measures for which no a priori predictions were made, two-tailed tests were conducted with Bonferroni corrections applied (the alpha level was divided by four for the NEO-FFI and by nine for the PVQ). To explore the relationships between scores, Pearson’s product–moment correlations were calculated for the scores, which were consistently associated with the scientist group in the above analyses. The results of all tests were considered statistically significant at a value of *p* < 0.05.

## Results

The means (with *SD*s) and correlations of all scores in the scientist and non-scientist groups are reported in Additional file 2: Table S2.

**Fig. 1:**
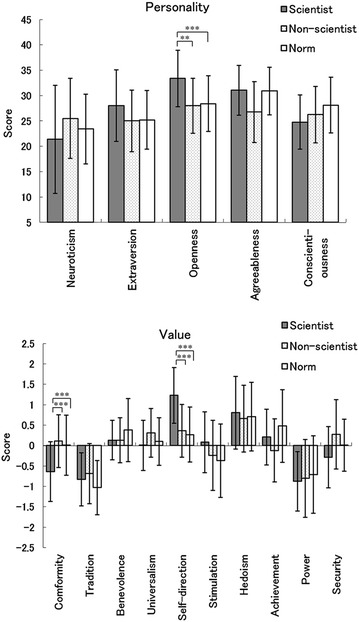
Mean (with *SD*) scores of personality (*upper*) and values (*lower*) in scientist group (scientist), non-scientist control group (non-scientist), and normative laypeople data (Norm). ***p < 0.001; **p < 0.01

In terms of personality traits (Fig. 1, upper), *t*-tests comparing the scientist and non-scientist groups revealed that the scientist group exhibited significantly higher scores on openness [*t*(48) = 2.78, *p* < 0.005]. There were no other significant differences in personality traits between the groups (*p*s > 0.1). The comparison between the scientist group and normative data also showed that scientists exhibited significantly higher scores on openness [*t*(355) = 4.28, *p* < 0.001]. There was a non-significant tendency for scientists to score lower measures of conscientiousness [*t*(356) = 2.28, *p* < 0.1]. There were no significant differences in other personality traits between the groups (*p*s > 0.1).

In terms of values (Fig. 1, lower), results from the *t* tests comparing the scientist and non-scientist groups revealed that the scientist group exhibited significantly higher scores on self-direction and lower scores on conformity [*t*(48) = 4.62 and 3.82, respectively, *p*s < 0.001]. There were no other significant differences between the groups (*p*s > 0.1). In the comparison between the scientist group and the normative data, the results also revealed that scientists exhibited significantly higher scores on self-direction and lower scores on conformity [*t*(186) = 6.53 and 4.00, respectively, *p*s < 0.001].

**Fig. 2:**
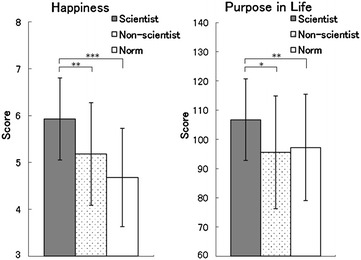
Mean (with *SD*) scores of subjective happiness (*left*) and purpose in life (*right*) in scientist group (scientist), non-scientist control group (non-scientist), and normative laypeople data (Norm). ***p < 0.001; ** *p* < 0.01; *p < 0.05

The scores for subjective happiness (Fig. 2, left) were higher in the scientist group across comparison between the scientist and non-scientist groups and between the scientists and normative data [*t*(48) = 2.60, *p* < 0.01; *t*(324) = 5.60, *p* < 0.001]. The scores for purpose in life (Fig. 2, right) were also higher in the scientist group in comparison with both the non-scientist group and the normative data [*t*(48) = 2.34, *p* < 0.05; *t*(185) = 2.45, *p* < 0.01].

Because there were group differences in the scores on openness, self-direction, subjective happiness, and purpose in life between scientists and non-scientists/laypeople, the relationships between these scores in scientists were further analyzed. The results showed a significant positive correlation between openness and self-direction (*r* = 0.59, *p* < 0.005). A significant positive correlation was also found between subjective happiness and purpose in life (*r* = 0.49, *p* < 0.05). There were no significant correlations of openness—subjective happiness, openness—purpose in life, self-direction—subjective happiness, or self-direction—purpose in life (|*r*| < 0.15, *p* > 0.1).

## Discussion

The analyses for personality traits showed that scientists consistently scored higher on openness compared with the non-scientist control group and with the normative data of laypeople. This result is consistent with previous studies on scientists’ personality traits, particularly those involved in creative endeavors (e.g., Grosul and Feist 2014). These data corroborate the notion that scientists exhibit intelligence and curiosity as primary personality characteristics.

More important, the values analyses revealed that scientists had stronger self-direction values compared with the non-scientist control group and with the normative data drawn from a population of laypeople. A high degree of self-direction in scientists is theoretically plausible, as this value corresponds to curiosity, creativity, and autonomous action (Schwartz 2012). This result confirms the traditional view that scientists’ primary motivations relate to curiosity, intellectual exploration, and autonomy (Polanyi 1958; Weber 1922). As a challenge to the traditional view, it has been proposed that other motivations, such as the desire to obtain prestige or monetary reward, may be more important to scientists, based on anecdotal records of scientists’ misconduct (Broad and Wade 1982). However, the results of this study do not support this view; scientists did not exhibit a reliably stronger inclination toward the value of power (i.e., placing importance on the pursuit of prestige, dominance, and material resources; Schwartz 2012) than non-scientists. The analyses additionally showed that scientists, compared with non-scientists, scored lower on conformity (i.e., placing importance on avoiding upsetting others and complying with expectations; Schwartz 2012). This result is consistent with the previous results that self-direction and conformity values are negatively correlated and located at opposite positions of the multivariate space (Schwartz et al. 2001). These findings can provide foundation for investigating other psychological characteristics of scientists. Schwartz’s 10 basic values model has been implemented in divergent lines of research, and has provided evidence regarding the associations between values and various behaviors (e.g., a positive association between self-direction values and center-left political attitudes; Schwartz 2006). To the best of my knowledge, this study provides the first evidence that scientists are primarily motivated by self-direction values.

Furthermore, the results demonstrated that scientists scored higher on subjective happiness and sense of purpose in life in comparison with the non-scientist control group and the normative data of laypeople. These results are consistent with previous investigations regarding mental health problems (e.g., Kessler et al. 2005). However, no study has investigated the subjective well-being of scientists. The results are also consistent with anecdotal evidence that great scientists of the past derived happiness and satisfaction from their scientific activities (e.g., Wolpert and Richards 1997). However, such data are qualitative and restricted only to the most-eminent scientists. The results from this study indicate that scientists, compared with non-scientists, experience higher levels of affective and eudaimonic subjective well-being. The data have interesting implications for understanding the psychological conditions of actual scientists, and provide a well-articulated description of an occupational option that can provide subjective well-being to those who are well suited to it.

The correlational analyses showed positive and moderate associations between openness and self-direction in scientists. Positive associations were also present between the subjective happiness and purpose in life variables. These results are consistent with the previous data that show associations between openness and self-direction (Parks-Leduc et al. 2015; Roccas et al. 2002; Vecchione et al. 2011) and between subjective happiness and purpose in life (French and Joseph 1999; Sato et al. 2015; Wnuk et al. 2012). In contrast, the personality traits and values did not correlate with the subjective well-being scores. These data suggest that openness traits and self-direction values in scientists are not directly related to subjective well-being, but indirectly connected via their behaviors and performance.

The results of this study may have practical implications. For example, during the recruitment of scientists, it may be beneficial to evaluate the personality traits and values of candidates in addition to their intellectual abilities. Furthermore, the results may also be useful in terms of improving management in scientific institutes. Because scientists exhibit strong self-directedness, which includes motivation to explore new ideas and perform autonomous action, scientists may be happy if they are provided with the opportunity to learn and to take initiative in their jobs. These findings are also important for scientific education. Since scientists’ lives tend to be happy and meaningful, they can recruit and subsequently educate their successors and instill them with confidence, thereby increasing their subjective well-being.

This study is subject to some limitations that suggest directions for future research. First, the study did not include performance measures, such as publication and citation counts (Feist 1997). Therefore, it remains unknown whether the values and subjective well-being scores found in this study could be associated with productivity and creativity. Several previous studies have reported that openness is positively related to scientists’ creativity levels (e.g., Grosul and Feist 2014; for a review, see Feist 2006c). Since this study showed a positive correlation between openness and self-direction in scientists, one may expect to see a positive association between self-direction values and creativity in scientists. Furthermore, ample evidence has shown that subjective well-being generally improves cognitive performance (for a review, see Lyubomirsky et al. 2005). It may be possible that subjective well-being is positively related to performance in scientists.

Second, because the sample of scientists was small and homogeneous, the generalizability of the findings may be limited. Specifically, all the data were collected from scientists at a single institute in Japan, who may share common institutional and cultural traits. Because all the scientists were associate or assistant professors, it would be informative to collect data from younger scientists, such as students who have not yet acquired their doctoral degrees, and older scientists, such as managers of departments, as these groups may exhibit different values and degrees of subjective well-being. Further investigation of the values and subjective well-being of other scientist samples represents an important matter for future research.

Third, a group of scientists was compared with a group of non-scientists in this study; such categorizations, however, may be too generic and it may be productive to divide these general groups into additional sub-groups in future research. For example, a previous study (Rawlings and Locarnini 2008) subdivided scientists into groups of physical scientists/mathematicians and biological scientists and found that the former group showed stronger autistic characteristics (i.e., attention to detail and poor imagination; Baron-Cohen et al. 2001) than the latter. These data suggest that there may be some differences among disciplinary sub-groups of scientists. To further understand scientists’ psychological characteristics, future studies should investigate such sub-groups with respect to their personality traits, values, and subjective well-being.

Finally, a promising direction for further investigation is the use of alternative scales for assessing scientists’ values, motivations, and subjective well-being based on different theoretical frameworks. For example, in the motivation literature, there are several theories related to motivation for cognitive activities, such as goal oriented theory (Dweck 1986; Nicholls 1984) and needs theory (McClelland et al. 1953). Specifically, in terms of the needs theory, humans have basic motivations for achievement, affiliation, and power. McClelland (1962) therefore hypothesized that scientists would have higher levels of achievement motivation. In the subjective well-being literature, alternative eudaimonic components were proposed, such as environmental mastery and personal growth (Ryff 1989). It is possible that scientists would show high scores on these measures of subjective well-being as well. The investigation into these alternative measures would improve our understanding of scientists’ psychological characteristics.

## Conclusions

The results of this study revealed that scientists, as compared with non-scientists, exhibited a higher degree of openness, self-direction, subjective happiness, and sense of purpose. These data indicate that scientists tend to possess personality traits and values that are well-suited to a career in science, and they also tend to acquire subjective well-being through their sense of vocation.

## Additional files

10.1186/s40064-016-2225-2 Description of personality traits and values.
10.1186/s40064-016-2225-2 Means (with *SD*s) and correlations of all scores in the scientist and non-scientist groups.

## References

[CR1] Baron-Cohen S, Wheelwright S, Skinner R, Martin J, Clubley E (2001). The autism-spectrum quotient (AQ): evidence from asperger syndrome/high-functioning autism, males and females, scientists and mathematicians. J Autism Dev Disord.

[CR2] Barrick MR, Mount MK (1991). The big five personality dimensions and job performance: a meta-analysis. Pers Psychol.

[CR3] Baumeister RF, Vohs KD, Aaker JL, Garbinsky EN (2013). Some key differences between a happy life and a meaningful life. J Pos Psychol.

[CR4] Broad W, Wade N (1982). Betrayers of the truth: Fraud and deceit in the halls of science.

[CR5] Crumbaugh JC, Maholick LT (1964). An experimental study in existentialism: the psychometric approach to Frankl’s concept of noogenic neurosis. J Clin Psychol.

[CR6] Dweck CS (1986). Motivational processes affecting learning. Am Psychol.

[CR7] Feist GJ (1997). Quantity, quality, and depth of research as influences on scientific eminence: is quantity most important?. Creat Res J.

[CR8] Feist GJ (1998). A meta-analysis of personality in scientific and artistic creativity. Pers Soc Psychol Rev.

[CR9] Feist GJ (2006). Psychology of science as a new subdiscipline in psychology. Curr Dir Psychol Sci.

[CR10] Feist GJ (2006). The past and future of the psychology of science. Rev Gen Psychol.

[CR11] Feist GJ (2006). How development and personality influence scientific thought, interest, and achievement. Rev Gen Psychol.

[CR12] Feist GJ, Gorman ME (1998). The psychology of science: review and integration of a nascent discipline. Rev Gen Psychol.

[CR13] French S, Joseph S (1999). Religiosity and its association with happiness, purpose in life, and self-actualization. Ment Health Relig Cult.

[CR14] Grosul M, Feist GJ (2014). The creative person in science. Psychol Aesthet Creat Arts.

[CR15] Holland JL (1985). Making vocational choices: A theory of vocational personalities and work environments.

[CR16] Houts AC, Gholson B, Shadish WR, Neimeyer RA, Houts AC (1989). Contributions of the psychology of science to metascience: A call for explorers. Psychology of science: Contributions to metascience.

[CR17] Jindal-Snape D, Snape JB (2006). Motivation of scientists in a government research institute: scientists’ perceptions and the role of management. Manag Decis.

[CR18] Kauppinen A (2013). Meaning and happiness. Philosophical Topics.

[CR19] Kessler RC, Berglund P, Demler O, Jin R, Merikangas KR, Walters EE (2005). Lifetime prevalence and age-ofonset distributions of DSM-IV disorders in the National Comorbidity Survey replication. Arch Gen Psychiatr.

[CR20] Kringelbach ML, Berridge KC (2010). The functional neuroanatomy of pleasure and happiness. Discov Med.

[CR21] Lounsbury JW, Foster N, Patel H, Carmody P, Gibson LW, Stairs DR (2012). An investigation of the personality traits of scientists versus nonscientists and their relationship with career satisfaction. R&D Manag.

[CR22] Ludwig A (1995). The price of greatness: resolving the creativity and madness.

[CR23] Lyubomirsky S, Lepper H (1999). A measure of subjective happiness: preliminary reliability and construct validation. Soc Indic Res.

[CR24] Lyubomirsky S, King L, Diener E (2005). The benefits of frequent positive affect: does happiness lead to success?. Psychol Bull.

[CR25] McClelland D, Gruber H, Terrell G, Wertheimer M (1962). On the psychodynamics of creative physical scientists. Contemporary approaches to creative thinking.

[CR26] McClelland D, Atkinson JW, Clark RA, Lowell EL (1953). The achievement motive.

[CR27] Nicholls JG (1984). Achievement motivation: conceptions of ability, subjective experience, task choice, and performance. Psychol Rev.

[CR28] Parks-Leduc L, Feldman G, Bardi A (2015). Personality traits and personal values: a meta-analysis. Pers Soc Psychol Rev.

[CR29] Polanyi M (1958). Personal knowledge: Towards a post-critical philosophy.

[CR30] Pytlik LA, Hemenover SH, Dienstbier RD (2002). What do we assess when we assess a Big 5 trait? A content analysis of the affective, behavioral, and cognitive processes represented in Big 5 personality inventories. Pers Soc Psychol Bull.

[CR31] Rawlings D, Locarnini A (2008). Dimensional schizotypy, autism, and unusual word associations in artists and scientists. J Res Pers.

[CR32] Roccas S, Sagiv L, Schwartz SH, Knafo A (2002). The Big Five personality factors and personal values. Pers Soc Psychol Bull.

[CR33] Russell B (1930). The conquest of happiness.

[CR34] Ryff CD (1989). Happiness is everything, or is it? Explorations on the meaning of psychological well-being. J Pers Soc Psychol.

[CR35] Sato F (1986). A study on purpose-in-lif test (PIL) I. Artes liberales.

[CR36] Sato W, Kochiyama T, Uono S, Kubota Y, Sawada R, Yoshimura S, Toichi M (2015). The structural neural substrate of subjective happiness. Sci Rep.

[CR37] Schwartz SH, Zanna M (1992). Universals in the content and structure of values: theory and empiric al tests in 20 countries. Advances in experimental social psychology (Vol. 25).

[CR38] Schwartz SH (2006). Basic human values: theory, measurement, and applications. Revue francaise de sociologie.

[CR39] Schwartz SH (2012). An overview of the Schwartz theory of basic values. Online Read Psychol Cult.

[CR40] Schwartz SH, Melech G, Lehmann A, Burgess S, Harris M (2001). Extending the cross-cultural validity of the theory of basic human values with a different method of measurement. J Cross Cult Psychol.

[CR41] Shadish WR, Houts AC, Gholson B, Neimeyer RA, Gholson B, Shadish WR, Neimeyer RA, Houts AC (1989). The psychology of science: An introduction. Psychology of science: contributions to metascience.

[CR42] Shimai S, Otake K, Utsuki N, Ikemi A, Lyubomirsky S (2004). Development of a Japanese version of the subjective happiness scale (SHS), and examination of its validity and reliability. Nippon Koshu Eisei Zasshi.

[CR43] Soldner T (2013) Personality, values, and cultural perceptions in the sojourner context: A new perspective on acculturation in Germany, Japan, and the US. Digitale dissertation, Humboldt-Universitat Berlin

[CR44] Vecchione M, Alessandri G, Barbaranelli C, Caprara G (2011). Higher-order factors of the big five and basic values: empirical and theoretical relations. Br J Psychol.

[CR45] Weber M (1922). Gesammelte Aufsätze zur Wissenschaftslehre.

[CR46] Wnuk M, Marcinkowsk JT, Fobair P (2012). The relationship of purpose in life and hope in shaping happiness among patients with cancer in Poland. J Psychosoc Oncol.

[CR47] Wolpert L, Richards A (1997). Passionate minds: the inner world of scientists.

