# Supplementary material for: Scientists’ personality, values, and well-being
Source: Springerplus. 2016 May 12;5:613. doi: 10.1186/s40064-016-2225-2 (PMC4864734; doi:10.1186/s40064-016-2225-2)
Supplement: Supplementary file 1 — 10.1186/s40064-016-2225-2 Description of personality traits and values. [file 40064_2016_2225_MOESM1_ESM.doc]

| Table S1. Description of personality traits and values. | | |
| --- | --- | --- |
| Scale/Component | | Description |
| Personality | |  |
|  | Neuroticism | Anxious, depressed, angry, embarrassed, emotional, worried, and insecure traits. |
|  | Extraversion | Sociable, gregarious, assertive, talkative, and active traits. |
|  | Openness | Imaginative, cultured, curious, original, broad-minded, intelligent, and artistically sensitive traits. |
|  | Agreeableness | Courteous, flexible, trusting, good-natured, cooperative, forgiving, soft-hearted, and tolerant traits. |
|  | Conscientiousness | Careful, thorough, responsible, organized, planful, hardworking, achievement-oriented, and persevering traits. |
| Value | |  |
|  | Conformity | Restraint of actions, inclinations, and impulses likely to upset or harm others and violate social expectations or norms. |
|  | Tradition | Respect, commitment and acceptance of the customs and ideas that traditional culture or religion provide the self. |
|  | Benevolence | Preservation and enhancement of the welfare of people with whom one is in frequent personal contact. |
|  | Universalism | Understanding, appreciation, tolerance and protection for the welfare of all people and for nature. |
|  | Self-direction | Independent thought and action-choosing, creating, exploring. |
|  | Stimulation | Excitement, novelty, and challenge in life. |
|  | Hedonism | Pleasure and sensuous gratification for oneself. |
|  | Achievement | Personal success through demonstrating competence according to social standards. |
|  | Power | Social status and prestige, control or dominance over people and resources. |
|  | Security | Safety, harmony and stability of society, of relationships, and of self. |
| Information is adapted from Barrick and Mount (1991) and Schwartz (2012) for personality and value, respectively. | | |
