# Supplementary material for: Scientists’ personality, values, and well-being
Source: Springerplus. 2016 May 12;5:613. doi: 10.1186/s40064-016-2225-2 (PMC4864734; doi:10.1186/s40064-016-2225-2)
Supplement: Supplementary file 2 — 10.1186/s40064-016-2225-2 Means (with SDs) and correlations of all scores in the scientist and non-scientist groups. [file 40064_2016_2225_MOESM2_ESM.doc]

| Table S2. Means (with *SD*s) and correlations of all scores in the scientist and non-scientist groups. | | | | | | | | | | | | | | | | | | | | | | | | | | | | | | | | | | | | |
| --- | --- | --- | --- | --- | --- | --- | --- | --- | --- | --- | --- | --- | --- | --- | --- | --- | --- | --- | --- | --- | --- | --- | --- | --- | --- | --- | --- | --- | --- | --- | --- | --- | --- | --- | --- | --- |
|  | Variable | *M* | *SD* | Correlation | | | | | | | | | | | | | | | | | | | | | | | | | | | | | | | | |
|  |  |  |  | 1 | | 2 | | 3 | | 4 | | 5 | | 6 | | 7 | | 8 | | 9 | | 10 | | 11 | | 12 | | 13 | | 14 | | 15 | | 16 | | |
| Scientist | |  |  |  |  |  |  |  |  |  |  |  |  |  |  |  |  |  |  |  |  |  |  |  |  |  |  |  |  |  |  |  |  | |  |  |
| 1 | Neuroticism | 21.39 | 10.65 |  |  |  |  |  |  |  |  |  |  |  |  |  |  |  |  |  |  |  |  |  |  |  |  |  |  |  |  |  |  | |  |  |
| 2 | Extraversion | 28.04 | 7.05 | -0.19 |  |  |  |  |  |  |  |  |  |  |  |  |  |  |  |  |  |  |  |  |  |  |  |  |  |  |  |  |  | |  |  |
| 3 | Openness | 33.38 | 5.56 | 0.03 |  | 0.28 |  |  |  |  |  |  |  |  |  |  |  |  |  |  |  |  |  |  |  |  |  |  |  |  |  |  |  | |  |  |
| 4 | Agreeableness | 31.04 | 4.89 | -0.61 | * | 0.30 |  | 0.08 |  |  |  |  |  |  |  |  |  |  |  |  |  |  |  |  |  |  |  |  |  |  |  |  |  | |  |  |
| 5 | Conscientiousness | 24.75 | 5.34 | -0.37 |  | -0.08 |  | -0.31 |  | -0.04 |  |  |  |  |  |  |  |  |  |  |  |  |  |  |  |  |  |  |  |  |  |  |  | |  |  |
| 6 | Comformity | -0.64 | 0.73 | -0.14 |  | 0.17 |  | -0.24 |  | 0.10 |  | 0.47 | * |  |  |  |  |  |  |  |  |  |  |  |  |  |  |  |  |  |  |  |  | |  |  |
| 7 | Tradition | -0.82 | 0.65 | 0.05 |  | -0.62 | * | -0.25 |  | -0.21 |  | 0.24 |  | 0.16 |  |  |  |  |  |  |  |  |  |  |  |  |  |  |  |  |  |  |  | |  |  |
| 8 | Benevolence | 0.13 | 0.48 | -0.21 |  | 0.63 | * | 0.01 |  | 0.15 |  | 0.26 |  | 0.35 |  | -0.48 | * |  |  |  |  |  |  |  |  |  |  |  |  |  |  |  |  | |  |  |
| 9 | Universalism | 0.01 | 0.61 | -0.37 |  | 0.06 |  | -0.08 |  | -0.03 |  | -0.06 |  | 0.17 |  | 0.14 |  | 0.30 |  |  |  |  |  |  |  |  |  |  |  |  |  |  |  | |  |  |
| 10 | Self-direction | 1.23 | 0.68 | -0.15 |  | -0.18 |  | 0.59 | * | 0.07 |  | -0.11 |  | -0.42 | * | -0.24 |  | -0.11 |  | -0.22 |  |  |  |  |  |  |  |  |  |  |  |  |  | |  |  |
| 11 | Stimulation | 0.08 | 0.75 | 0.29 |  | 0.35 |  | 0.52 | * | 0.21 |  | -0.56 | * | -0.30 |  | -0.47 | * | -0.06 |  | -0.45 | * | 0.22 |  |  |  |  |  |  |  |  |  |  |  | |  |  |
| 12 | Hedoism | 0.80 | 0.89 | 0.09 |  | 0.38 |  | 0.45 | * | 0.08 |  | -0.30 |  | -0.06 |  | -0.37 |  | 0.45 | * | 0.08 |  | 0.09 |  | 0.29 |  |  |  |  |  |  |  |  |  | |  |  |
| 13 | Achievement | 0.21 | 0.68 | 0.37 |  | -0.06 |  | 0.18 |  | -0.05 |  | -0.31 |  | -0.52 | * | -0.16 |  | -0.46 | * | -0.51 | * | 0.16 |  | 0.42 |  | -0.32 |  |  |  |  |  |  |  | |  |  |
| 14 | Power | -0.88 | 0.73 | 0.21 |  | -0.10 |  | -0.28 |  | -0.33 |  | -0.23 |  | -0.49 | * | -0.10 |  | -0.11 |  | -0.17 |  | -0.08 |  | 0.09 |  | -0.19 |  | 0.26 |  |  |  |  |  | |  |  |
| 15 | Security | -0.28 | 0.75 | -0.08 |  | -0.01 |  | -0.34 |  | -0.24 |  | 0.55 | * | 0.41 | * | 0.33 |  | 0.02 |  | 0.28 |  | -0.45 | * | -0.62 | * | -0.40 |  | -0.17 |  | -0.28 |  |  |  | |  |  |
| 16 | Happiness | 5.93 | 0.88 | -0.69 | * | 0.39 |  | 0.14 |  | 0.44 | * | 0.11 |  | 0.35 |  | -0.05 |  | 0.30 |  | 0.40 |  | 0.11 |  | -0.15 |  | 0.16 |  | -0.45 | * | -0.49 | * | 0.06 |  | |  |  |
| 17 | Purpose in Life | 106.75 | 13.99 | -0.77 | * | 0.36 |  | -0.11 |  | 0.60 | * | 0.11 |  | 0.05 |  | -0.43 | * | 0.18 |  | -0.04 |  | 0.14 |  | 0.06 |  | -0.05 |  | -0.24 |  | -0.03 |  | -0.20 |  | | 0.49 | * |
| Non-scientist | |  |  |  |  |  |  |  |  |  |  |  |  |  |  |  |  |  |  |  |  |  |  |  |  |  |  |  |  |  |  |  |  | |  |  |
| 1 | Neuroticism | 25.27 | 7.87 |  |  |  |  |  |  |  |  |  |  |  |  |  |  |  |  |  |  |  |  |  |  |  |  |  |  |  |  |  |  | |  |  |
| 2 | Extraversion | 26.69 | 6.05 | -0.20 |  |  |  |  |  |  |  |  |  |  |  |  |  |  |  |  |  |  |  |  |  |  |  |  |  |  |  |  |  | |  |  |
| 3 | Openness | 29.04 | 5.43 | 0.44 | * | 0.10 |  |  |  |  |  |  |  |  |  |  |  |  |  |  |  |  |  |  |  |  |  |  |  |  |  |  |  | |  |  |
| 4 | Agreeableness | 29.38 | 5.97 | -0.05 |  | 0.41 | * | -0.06 |  |  |  |  |  |  |  |  |  |  |  |  |  |  |  |  |  |  |  |  |  |  |  |  |  | |  |  |
| 5 | Conscientiousness | 27.58 | 5.56 | -0.31 |  | 0.09 |  | -0.02 |  | 0.13 |  |  |  |  |  |  |  |  |  |  |  |  |  |  |  |  |  |  |  |  |  |  |  | |  |  |
| 6 | Comformity | 0.11 | 0.65 | 0.03 |  | 0.03 |  | -0.31 |  | 0.38 |  | 0.12 |  |  |  |  |  |  |  |  |  |  |  |  |  |  |  |  |  |  |  |  |  | |  |  |
| 7 | Tradition | -0.69 | 0.73 | 0.04 |  | -0.55 | * | -0.44 | * | -0.12 |  | -0.34 |  | 0.01 |  |  |  |  |  |  |  |  |  |  |  |  |  |  |  |  |  |  |  | |  |  |
| 8 | Benevolence | 0.13 | 0.55 | -0.17 |  | -0.02 |  | 0.12 |  | 0.23 |  | 0.24 |  | -0.14 |  | -0.13 |  |  |  |  |  |  |  |  |  |  |  |  |  |  |  |  |  | |  |  |
| 9 | Universalism | 0.31 | 0.60 | 0.14 |  | -0.16 |  | 0.11 |  | -0.02 |  | 0.55 | * | 0.01 |  | -0.32 |  | -0.09 |  |  |  |  |  |  |  |  |  |  |  |  |  |  |  | |  |  |
| 10 | Self-direction | 0.36 | 0.64 | -0.33 |  | 0.02 |  | 0.14 |  | -0.05 |  | 0.25 |  | 0.02 |  | -0.48 | * | 0.11 |  | 0.34 |  |  |  |  |  |  |  |  |  |  |  |  |  | |  |  |
| 11 | Stimulation | -0.24 | 0.86 | 0.08 |  | 0.12 |  | 0.35 |  | -0.25 |  | -0.22 |  | -0.29 |  | -0.29 |  | -0.21 |  | -0.13 |  | 0.22 |  |  |  |  |  |  |  |  |  |  |  | |  |  |
| 12 | Hedoism | 0.66 | 0.82 | 0.21 |  | 0.25 |  | 0.20 |  | 0.11 |  | -0.54 | * | 0.07 |  | -0.13 |  | -0.35 |  | -0.26 |  | -0.02 |  | 0.21 |  |  |  |  |  |  |  |  |  | |  |  |
| 13 | Achievement | -0.12 | 0.77 | -0.20 |  | 0.11 |  | -0.04 |  | 0.00 |  | 0.30 |  | -0.46 | * | -0.10 |  | 0.40 | * | 0.15 |  | -0.11 |  | -0.27 |  | -0.62 | * |  |  |  |  |  |  | |  |  |
| 14 | Power | -0.80 | 0.96 | 0.05 |  | 0.24 |  | 0.10 |  | -0.15 |  | -0.29 |  | -0.42 | * | 0.08 |  | 0.27 |  | -0.60 | * | -0.48 | * | -0.10 |  | -0.02 |  | 0.34 |  |  |  |  |  | |  |  |
| 15 | Security | 0.27 | 0.85 | 0.05 |  | -0.18 |  | -0.26 |  | 0.03 |  | 0.26 |  | 0.43 | * | 0.23 |  | -0.57 | * | 0.27 |  | -0.23 |  | -0.32 |  | -0.10 |  | -0.26 |  | -0.45 | * |  |  | |  |  |
| 16 | Happiness | 5.18 | 1.09 | -0.62 | * | 0.49 | * | -0.02 |  | 0.09 |  | 0.15 |  | -0.09 |  | -0.19 |  | -0.01 |  | -0.02 |  | 0.44 | * | -0.03 |  | -0.01 |  | 0.16 |  | 0.00 |  | -0.19 |  | |  |  |
| 17 | Purpose in Life | 95.50 | 19.31 | -0.55 | * | 0.54 | * | 0.15 |  | 0.20 |  | 0.47 | * | 0.08 |  | -0.53 | * | 0.03 |  | 0.28 |  | 0.54 | * | 0.05 |  | -0.12 |  | 0.15 |  | -0.26 |  | -0.01 |  | | 0.77 | * |
| * *p* < .05. | | | | | | | | | | | | | | | | | | | |  |  |  |  |  |  |  |  |  |  |  |  |  |  | |  |  |
